# Supplementary material for: De novo variants in the non-coding spliceosomal snRNA gene RNU4-2 are a frequent cause of syndromic neurodevelopmental disorders
Source: medRxiv. 2024 Apr 9:2024.04.07.24305438. Preprint. [Version 2] doi: 10.1101/2024.04.07.24305438 (PMC11030480; doi:10.1101/2024.04.07.24305438)
Supplement: Supplement 2 [file NIHPP2024.04.07.24305438v2-supplement-2.pdf]

medRxiv preprint doi: https://doi.org/10.1101/2024.04.07.24305438; this version posted April 9, 2024. The copyright holder for this preprint (which was not certified by peer review) is the author/funder, who has granted medRxiv a license to display the preprint in perpetuity.
It is made available under a CC-BY 4.0 International license .

## Supplementary Figures

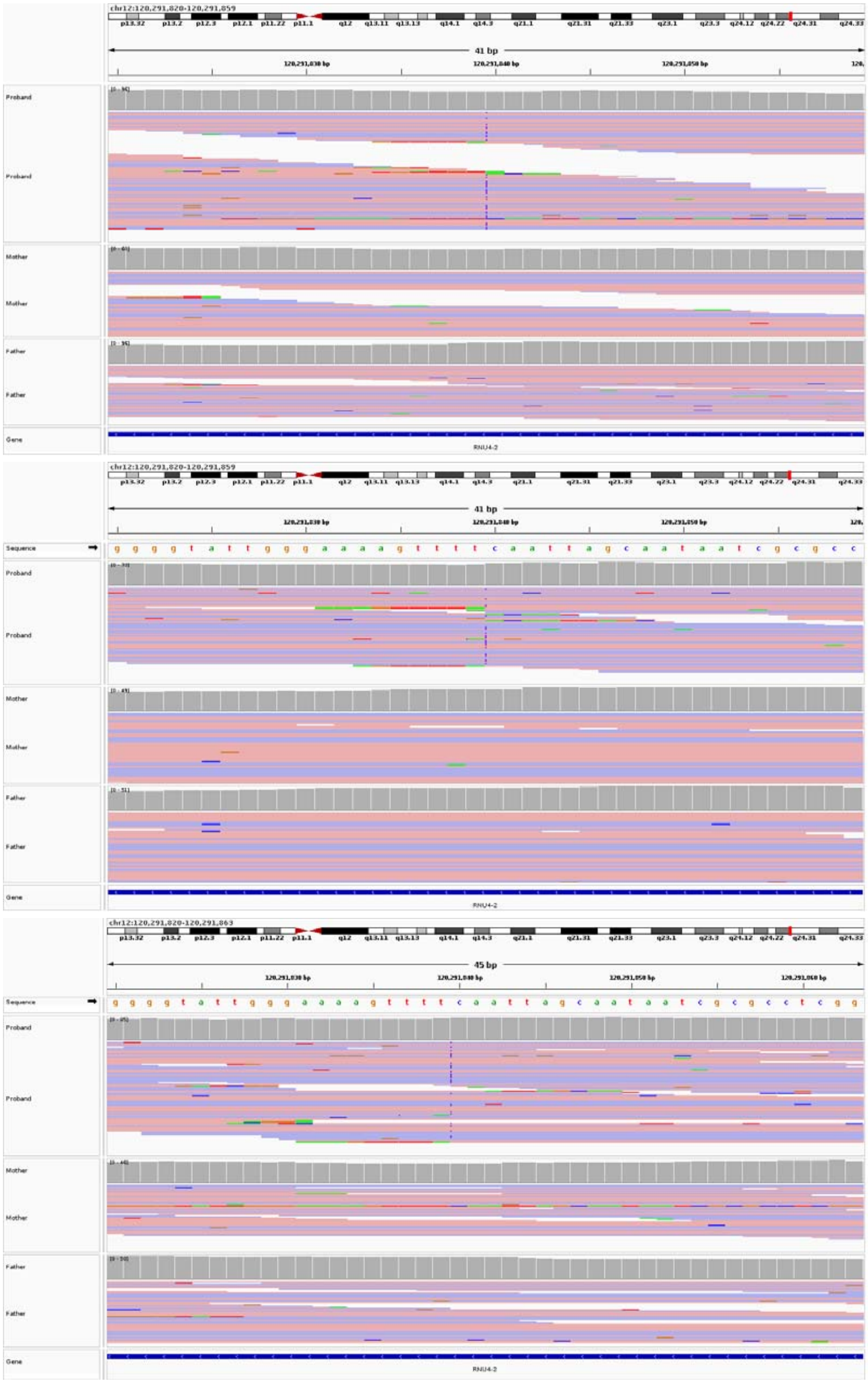

medRxiv preprint doi: https://doi.org/10.1101/2024.04.07.24305438; this version posted April 9, 2024. The copyright holder for this preprint (which was not certified by peer review) is the author/funder, who has granted medRxiv a license to display the preprint in perpetuity.
It is made available under a CC-BY 4.0 International license .

**Supplementary Figure 1**: Example IGV plots of the region surrounding the n.64_65insT variant in three trios.

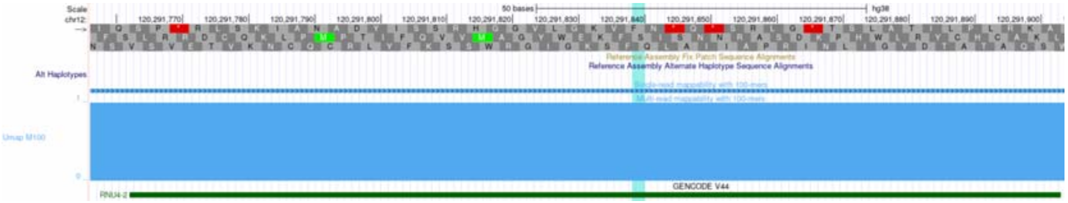


**Supplementary Figure 2**: Screenshot from the UCSC Genome Browser showing high mappability for 100-mers across the *RNU4-2* gene.

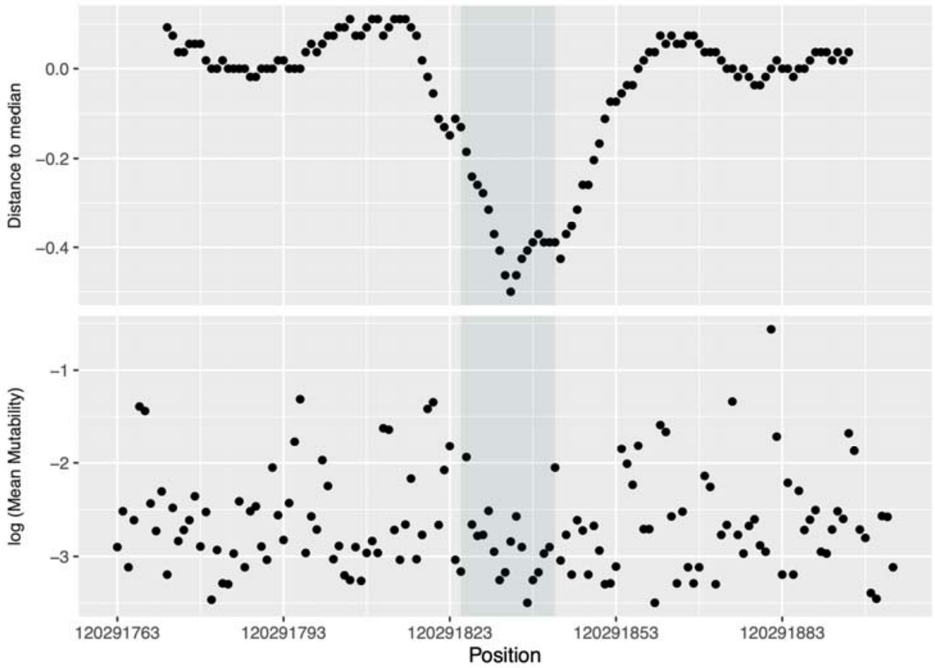


**Supplementary Figure 3**: (top) Distance to the median proportion of all possible SNVs that are observed in the UK Biobank in 18 bp sliding windows across the length of RNU4-2. A clear region of depletion compared to the rest of the gene is observed in the centre. (bottom) Log transformation of the mean Roulette[45] mutability across the 3 possible SNVs within a site.

medRxiv preprint doi: https://doi.org/10.1101/2024.04.07.24305438; this version posted April 9, 2024. The copyright holder for this preprint
(which was not certified by peer review) is the author/funder, who has granted medRxiv a license to display the preprint in perpetuity.
It is made available under a CC-BY 4.0 International license .

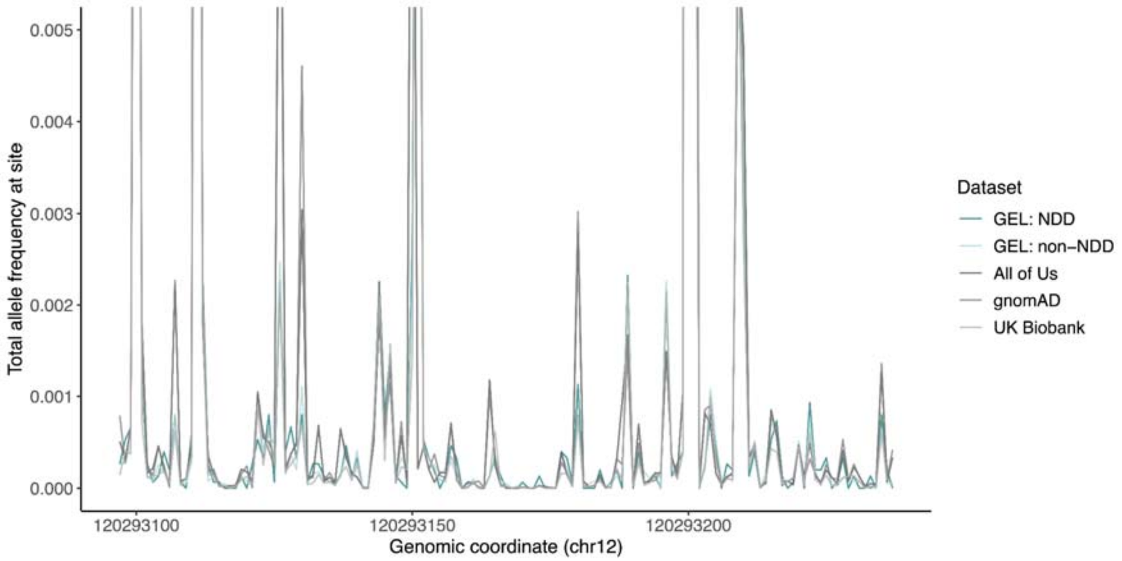


**Supplementary Figure 4**: Total allele frequency at each site of *RNU4-1* in five datasets. In contrast to *RNU4-2* (Figure 2a), variants in *RNU4-1* have higher allele frequencies. A similar region of depletion is seen in the centre of *RNU4-1* (quantified in Figure 4), but this is not enriched for variants in GEL NDD or non-NDD individuals.

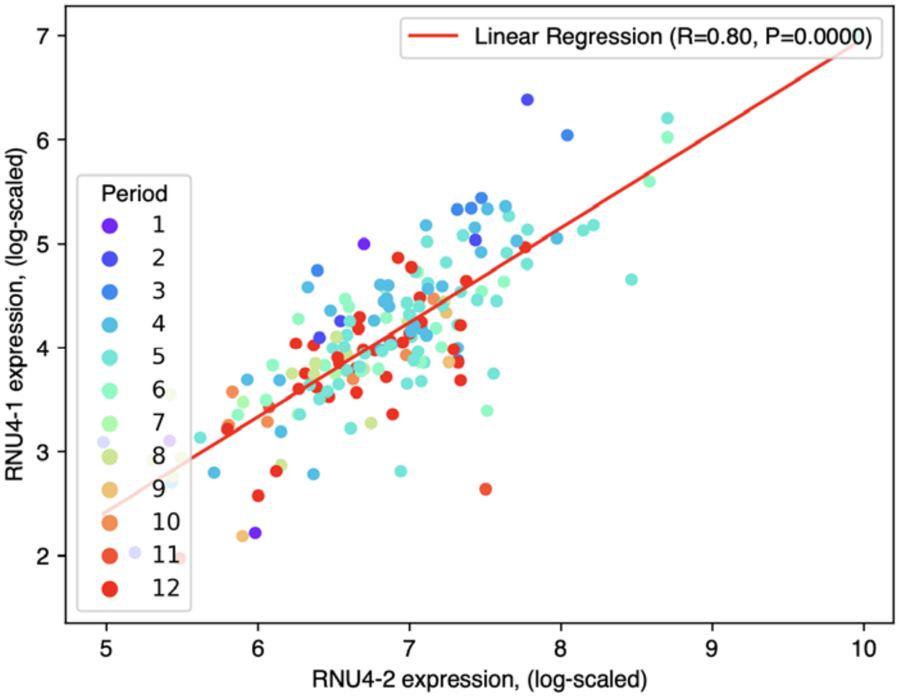


**Supplementary Figure 5**: Correlation between *RNU4-1* and *RNU4-2* expression in RNA-seq data from human cortex across prenatal and postnatal development from BrainVar[28].

medRxiv preprint doi: https://doi.org/10.1101/2024.04.07.24305438; this version posted April 9, 2024. The copyright holder for this preprint (which was not certified by peer review) is the author/funder, who has granted medRxiv a license to display the preprint in perpetuity.
It is made available under a CC-BY 4.0 International license .

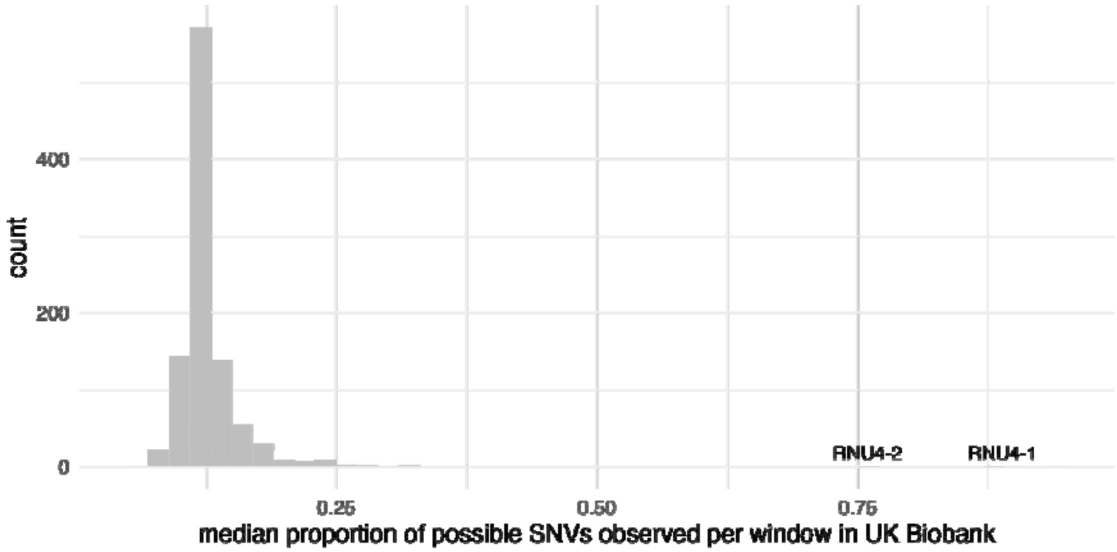


**Supplementary Figure 6**: Median proportion of possible SNVs observed in UK Biobank per 18 bp window across 1,000 intergenic regions on chromosome 12 (grey) and *RNU4-1*, *RNU4-2* (teal).

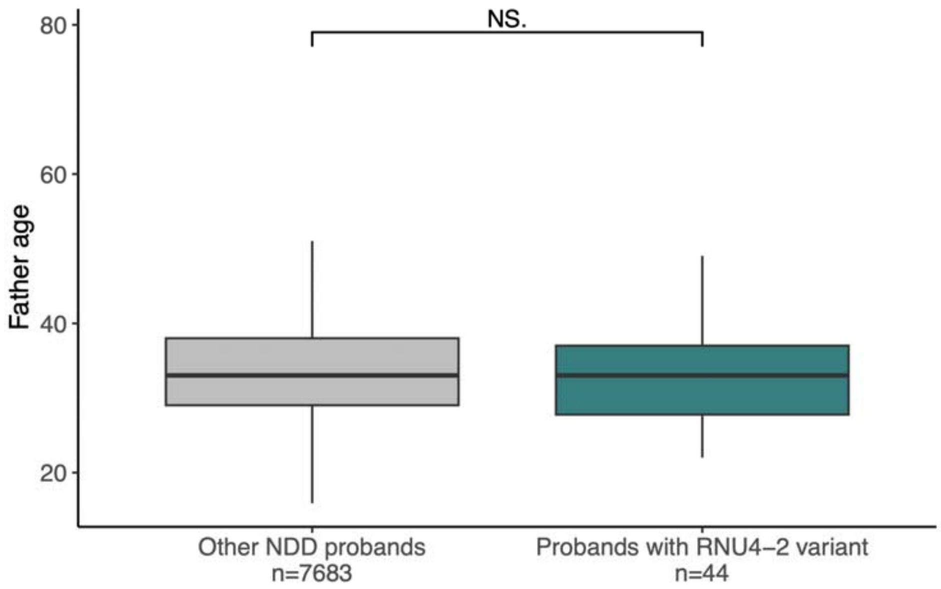


**Supplementary Figure 7**: Comparison of paternal age for probands with fathers recruited into GEL.

## Supplementary Tables

**Supplementary Table 1**: The number of probands with the n.64_65insT variant and all other individuals with NDD with HPO terms corresponding to phenotypes observed in ≥ 5 individuals compared to all other NDD probands. These data are plotted in Figure 1a. A P-value threshold of $2.94 \times 10^{-3}$ was used to assess statistical significance (Bonferroni adjusted for 17 tests).

**Supplementary Table 2**: ICD10 and ICD9 codes for individuals with single base pair insertions between codons 64 and 65 of *RNU4-2* and *RNU4-1* in the UK Biobank.

medRxiv preprint doi: https://doi.org/10.1101/2024.04.07.24305438; this version posted April 9, 2024. The copyright holder for this preprint (which was not certified by peer review) is the author/funder, who has granted medRxiv a license to display the preprint in perpetuity.
It is made available under a CC-BY 4.0 International license .

**Supplementary Table 3**: Outliers predicted by OUTRIDER and FRASER2 in RNA-seq data for five individuals with *RNU4-2* variants compared to 5,409 controls. A P-value threshold of 0.017 was used to assess statistical significance (Bonferroni adjusted for 3 tests).

**Supplementary Table 4**: Detailed clinical information for 25 individuals with RNU4-2 variants. SNVs are highlighted in pink, and the individual with an alternate indel in blue. Blank spaces indicate that data were not provided.

**Supplementary Table 5**: Detailed phenotypic information for individuals with the n.64_65insT variant across cohorts.

**Supplementary Table 6**: Mean expression of U4 genes in prefrontal cortex across all samples in BrainVar.

**Supplementary Table 7**: Genomic coordinates of, and burden testing results for snRNA genes.

**Supplementary Table 8**: Sub-regions of snRNA genes identified as depleted of variation and burden testing results in these regions.
